# Supplementary material for: Primary Cardiac Sarcomas: Clinical Characteristics, Management, and Outcomes at a Spanish National Reference Center
Source: Cancers (Basel). 2025 Dec 10;17(24):3947. doi: 10.3390/cancers17243947 (PMC12730294; doi:10.3390/cancers17243947)
Supplement: Supplementary file 1 [file cancers-17-03947-s001.zip › cancers-3985839-supplementary.pdf]

Supplementary Material

Supplementary Table S1. Comprehensive histopathologic and molecular data collected for each individual patient.

Patient	Definitive Diagnosis	Histopathology	IHC	Molecular Testing
#1	Angiosarcoma	Spindle-cell proliferation of intermediate cellular density forming well-defined vascular slits with red blood cell extravasation. Cells have poorly defined eosinophilic cytoplasm and generally isomorphic spindle-shaped nuclei with dense chromatin; occasional mitoses are observed. Dense collagenous stroma with areas of interstitial hemorrhage. No epithelial or heterologous differentiation.	**Positive:**<br>CD31, CD34, factor VIII, and podoplanin (weak and focal), C-MYC<br>**Negative:**<br>TFE3, smooth muscle actin (ML), cytokeratin AE1–AE3, STAT6, S100<br>**Ki-67**<br>70%	Not available
#2	Undifferentiated pleomorphic sarcoma, FNCLCC Grade 2	Hypercellular mesenchymal tumor composed mainly of spindle cells with poorly defined eosinophilic cytoplasm and spindle-shaped nuclei with fine chromatin, some with prominent nucleoli, within a dense collagenous stroma with minor myxoid change. Scattered pleomorphic cells. No heterologous or epithelial differentiation. Focally dilated hemangiopericytoma-like vessels. 2 mitoses/10 HPF.	**Positive:**<br>S100 (focal), TRK, MDM2<br>**Negative:**<br>Actin ML, HHF35 actin, desmin, myogenin, CD34, CD31, HMB-45, Melan-A, SOX10, cytokeratin AE1/AE3, CD45, c-KIT, STAT6, factor XIIIa, factor VIII, podoplanin<br>**Ki-67**<br>30%	**FISH:**<br>No EWSR1 or NTRK amplification; MDM2 amplification detected
#3	Intimal sarcoma	Neoplastic mesenchymal proliferation growing in sheets with marked cellularity. Composed of intermediate-sized spindle cells in intersecting fascicles, with poorly defined eosinophilic cytoplasm and elongated to ovoid hyperchromatic nuclei; scattered pleomorphic cells within a collagenous stroma with myxoid areas and focal osteoid formation. Peripheral vascular wall tissue observed. Six mitoses/10 HPF. No necrosis identified.	**Positive:**<br>HHF35 Actin, Smooth Muscle Actin (ML), H-Caldesmon, MDM2<br>**Negative:**<br>Desmin	Not available
#4	Angiosarcoma	Mesenchymal neoplasm with extensive hemorrhage, composed of spindle cells in a solid pattern, forming anastomosing vascular channels and fascicular areas with focal intraluminal papillary structures. Nuclei enlarged and hyperchromatic with mitotic figures; 18 mitoses/10 HPF. Foci of hemorrhagic necrosis affecting >50% of the submitted material.	**Positive:**<br>CD31, CD34, ERG<br>**Negative:**<br>CK AE1–AE3, EMA, Caldesmon, SMA, Myogenin, Desmin, Calretinin, MDM2, D2-40, S100, STAT 6, FOSB, CAMTA-1<br>**Ki67:**<br>30%	**Mutation panel[1]:**<br>Negative<br>**Fusion panel[2]:**<br>Negative

#5	Undifferentiated pleomorphic sarcoma, FNCLCC Grade 3	Hypercellular mesenchymal tumor composed mainly of spindle cells with poorly defined eosinophilic cytoplasm and spindle-shaped nuclei, alternating with epithelioid and occasional pleomorphic cells, within a dense collagenous stroma with minor myxoid areas. Foci of necrosis and frequent atypical mitoses. No heterologous or epithelial differentiation. Scattered dilated hemangiopericytoma-like vessels at the periphery. 25 mitoses/10 HPF.	**Positive:**<br>Vimentin, FLI1, TLE, P53<br><br>**Negative:**<br>Smooth muscle actin (ML), HHF35 actin, desmin, myogenin, CD34, CD68, CK AE1/AE3, c-KIT, MDM2, CDK4, EMA, SS18-SSX, STAT6, MUC4<br><br>**Ki-67:**<br>40%	**Mutation panel[1]:**<br>Negative<br><br>**Fusion panel[2]:**<br>Negative
#6	Angiosarcoma	Predominantly spindle-cell mesenchymal proliferation in a solid, infiltrative pattern within fibrous tissue. Cells with poorly defined eosinophilic cytoplasm and elongated nuclei, some enlarged and hyperchromatic. 4 mitoses/10 HPF.	**Positive:**<br>CD31, CD34, ERG, smooth muscle actin (focal), c-MYC (focal)<br><br>**Negative:**<br>S100, MDM2, p53, Cyclin D1, HHV8, CAMTA-1<br><br>**Ki-67:**<br>25%	Not available
#7	Angiosarcoma	Neoplasm with predominantly solid, infiltrative growth, interstitial hemorrhage, and focal anastomosing vascular structures lined by atypical cells. Cells with high N:C ratio, oval nuclei with anisokaryosis, irregular nuclear membranes, heterogeneous chromatin, occasional nucleoli. Frequent mitoses. No necrosis observed.	**Positive:**<br>ERG, CD31, podoplanin, WT1 (cytoplasmic)<br><br>**Negative:**<br>CK AE1/AE3, CD45, CD20, CD3, CD79a, SALL4, calretinin, S100, SOX10, Melan-A<br><br>**Ki-67:**<br>>50% in hotspots	**Mutation panel[1]:**<br>Pathogenic variant TP53 (NM_000546.5): c.817C>T, p.Arg273Cys.<br><br>**Fusion panel[2]:**<br>Negative
#8	Undifferentiated round cell sarcoma	Densely cellular neoplasm with syncytial growth, composed of small cells with poorly defined borders and oval hyperchromatic nuclei. Frequent mitoses and areas of tumor necrosis. No reference structures, vascular, or perineural invasion observed.	**Positive:**<br>CD99, WT1, Bcl-2, BRG1/SMARCA4<br><br>**Negative:**<br>CK AE1/AE3, EMA, CD34, Myogenin, Desmin, Smooth Muscle Actin (ML), S100, c-KIT, NKX2.2, SS18, TTF1, NUT<br><br>**Ki-67:**<br>90%	**Fusion panel[2]:**<br>Negative
#9	Angiosarcoma	Collagenous tissue infiltrated by poorly differentiated spindle-cell neoplasm of variable cellularity, with frequent interstitial red blood cells. Cells with spindle-shaped nuclei and fine chromatin, some atypical. 2 mitoses/10 HPF. No epithelial differentiation observed.	**Positive:**<br>CD31, CD34, ERG<br><br>**Negative:**<br>Podoplanin, MDM2, SS18, CAMTA-1, HHV8	Not available

#10	Angiosarcoma	Mesenchymal proliferation with pseudolobular/solid pattern, non-encapsulated, within pulmonary parenchyma. Composed of spindle cells with poorly defined, weakly eosinophilic cytoplasm and elongated to oval nuclei with dense, generally isomorphic chromatin. 3 mitoses/10 HPF; no necrosis. Focal cystic areas and vessels with flat endothelium containing blood and fibrous septa. No collagenous or chondroid stroma, no epithelioid differentiation.	**Positive:**<br>CD31, CD34, Factor VIII, FLI-1<br><br>**Negative:**<br>Podoplanin, GLUT1, C-MYC, CK AE1/AE3, Smooth Muscle Actin (ML), S100, TTF-1, HHV8<br><br>**Ki-67:**<br>60%	Not available
#11	Undifferentiated pleomorphic/spindle cell sarcoma, FNCLCC Grade 3	Densely cellular mesenchymal proliferation of spindle and pleomorphic cells with poorly defined eosinophilic cytoplasm and nuclei with dense or fine chromatin, some with prominent nucleoli. Cells arranged in solid sheets and irregular fascicles over dense collagenous stroma. Areas of necrosis and mitoses (8/10 HPF), some atypical. No epithelial or heterologous differentiation.	**Positive:**<br>HHF35 actin, ML actin, CK AE1/AE3 (focal)<br><br>**Negative:**<br>MDM2, S100, SOX10, desmin, H-caldesmon, CD31, CD34, myogenin, calretinin, desmin<br><br>**Ki-67:**<br>80%	**Mutation panel[1]:**<br>Negative<br><br>**Fusion panel[2]:**<br>Negative
#12	Undifferentiated pleomorphic sarcoma, FNCLCC Grade 3	Pleomorphic mesenchymal proliferation with areas of epithelioid morphology on myxoid stroma and extensive interstitial edema. Cells with broad, poorly defined eosinophilic cytoplasm and pleomorphic nuclei with dense chromatin; mitoses, mostly atypical (18/10 HPF). Extensive multifocal necrosis (~40% of tumor). No epithelial or heterologous differentiation. Tumor infiltrates included arterial vessel.	**Positive:**<br>HHF35 actin (focal)<br><br>**Negative:**<br>ML actin, desmin, myogenin, S100, SOX10, MDM2, CK AE1/AE3, CD31, ERG, Factor VIII<br><br>**Ki-67:**<br>20%	Not available

IHC = Immunohistochemistry. FNCLCC = *Fédération Nationale des Centres de Lutte Contre le Cancer*. [1] **Mutation panel:** Extraction and purification of genomic DNA and RNA from a formalin-fixed paraffin-embedded tissue sample. Analysis of a gene panel using capture-based enrichment (Custom Solid Tumor Solution, Sophia Genetics) followed by sequencing (MiSeq, Illumina). Bioinformatic analysis was performed using Sophia Genetics' software and algorithms (Sophia DDM), as well as the IGV sequence viewer. Alignment was done against the GRCh37/hg19 reference genome. This methodology allows the detection of point mutations and small insertions and deletions. Only pathogenic or likely pathogenic variants are reported. Genomic alteration analysis was performed using a panel covering various regions of genes related to solid tumors (AKT1, ALK, ARID1A, BRAF, BRCA1, BRCA2, CDK4, CDKN2A, CTNNB1, DDR2, DICER1, EGFR, ERBB2, ERBB4, FBXW7, FGFR1, FGFR2, FGFR3, FOXL2, GNA11, GNAQ, GNAS, H3F3A, H3F3B, HIST1H3B, HRAS, IDH1, IDH2, KIT, KMT2A, KMT2D, KRAS, MAP2K1, MAP2K2, MET, MTOR, MYOD1, NRAS, PDGFRA, PIK3CA, PTPN11, RAC1, RAF1, RET, ROS1, SF3B1, SMAD4, TERT, TGFBR2, and TP53). [2] **Fusion panel:** Extraction and purification of genomic RNA from a formalin-fixed paraffin-embedded tissue sample. Analysis of a gene panel using amplicon-based enrichment (Archer FusionPlex Sarcoma Panel) followed by sequencing (MiSeq, Illumina). Alignment against the GRCh37/hg19 reference genome. Variant analysis was performed on the Archer platform. This methodology allows the detection of gene fusions in sarcoma-related genes (ALK, CAMTA1, CCNB3, CIC, EPC1, EWSR1, FOXO1, FUS, GLI1, HMGA2, JAZF1, MEAF6, MKL2, NCOA2, NTRK3, PDGFB, PLAG1, ROS1, SS18, STAT6, TAF15, TCF12, TFE3, TFG, USP6, YWHAE). Limitations: Detection of gene fusions may be limited by RNA quality and concentration.

Supplementary Table S2. Most frequently administered chemotherapy regimens in the cohort (detailed).

**Anthracycline-Containing Regimens**	
**Doxorrubicin**	Doxorubicin 75 mg/m², administered intravenously every 21 days.<br>Limited to six cycles.
**Epirrubicin-ifosfamide**	Epirubicin 60 mg/m², administered intravenously on days 1 and 2 of each 21-day cycle, plus ifosfamide 3 g/m², administered intravenously on days 1–3 of each 21-day cycle.<br>Limited to six cycles.
**VDC/IE**	Alternating cycles of vincristine 1.5 mg/m² (capped at 2 mg) + cyclophosphamide 1200 mg/m² + doxorubicin 75 mg/m² on day 1 IV; and etoposide 100 mg/m² + ifosfamide 1800 mg/m² on days 1–5 IV; cycles every 14 days.<br>Limited to 14 cycles; maximum of 6 cycles containing doxorubicin.
**Taxane-containing regimens**	
**Weekly paclitaxel**	Paclitaxel 80 mg/m² administered intravenously on days 1, 8, and 15; cycles every 28 days.
**Docetaxel-Gemcitabine**	Gemcitabine 900 mg/m² administered intravenously on days 1 and 8 + docetaxel 75 mg/m² administered intravenously on day 8; cycles every 21 days.
**Gemcitabine-containing regimens**	
**Gemcitabine-Dacarbazine**	Gemcitabine 1800 mg/m² administered intravenously + dacarbazine 500 mg/m² administered intravenously; cycles every 14 days.
**Docetaxel-Gemcitabine**	Same regimen as previously described.
